# SCM-198 inhibits microglial overactivation and attenuates Aβ_1-40_-induced cognitive impairments in rats via JNK and NF-кB pathways

**DOI:** 10.1186/s12974-014-0147-x

**Published:** 2014-08-19

**Authors:** Zhen-Yi Hong, Xue-Ru Shi, Kai Zhu, Ting-Ting Wu, Yi-Zhun Zhu

**Affiliations:** Department of Pharmacology, Research Building, School of Pharmacy, Fudan University, 826 Zhangheng Road, Zhanjiang Hi-Tech Park, Pudong New District, Shanghai, 201203 China; Department of Pharmacology, National University of Singapore, Singapore, Singapore

**Keywords:** SCM-198, Microglia, Lipopolysaccharide, β-Amyloid_1–40_, Alzheimer’s disease, Morris water maze, Co-culture, Neuroinflammation, Primary neuron

## Abstract

**Background:**

Neuroinflammation mediated by overactivated microglia plays a key role in many neurodegenerative diseases, including Alzheimer’s disease (AD). In this study, we investigated for the first time the anti-neuroinflammatory effects and possible mechanisms of SCM-198 (an alkaloid extracted from *Herbaleonuri*), which was previously found highly cardioprotective, both *in vitro* and *in vivo*.

**Methods:**

For *in vitro* experiments, lipopolysaccharide (LPS) or β-amyloid_1-40_ (Aβ_1-40_) was applied to induce microglial overactivation. Proinflammatory mediators were measured and activations of NF-κB and mitogen-activated protein kinases’ (MAPKs) pathways were investigated. Further protective effect of SCM-198 was evaluated in microglia-neuron co-culture assay and Sprague-Dawley (SD) rats intrahippocampally-injected with Aβ_1-40_.

**Results:**

SCM-198 reduced expressions of nitric oxide (NO), TNF-α, IL-1β and IL-6 possibly via, at least partially, inhibiting c-Jun N-terminal kinase (JNK) and NF-κB signaling pathways in microglia. Co-culture assay showed that activated microglia pretreated with SCM-198 led to less neuron loss and decreased phosphorylation of tau and extracellular signal-regulated kinase (ERK) in neurons. Besides, SCM-198 also directly protected against Aβ_1-40_-induced neuronal death and lactate dehydrogenase (LDH) release in primary cortical neurons. For *in vivo* studies, SCM-198 significantly enhanced cognitive performances of rats 12 days after intrahippocampal injections of aged Aβ_1-40_ peptides in the Morris water maze (MWM), accompanied by less hippocampal microglial activation, decreased synaptophysin loss and phosphorylation of ERK and tau. Co-administration of donepezil and SCM-198 resulted in a slight cognitive improvement in SD rats 50 days after intrahippocampal injections of aged Aβ_1-40_ peptides as compared to only donepezil or SCM-198 treated group.

**Conclusions:**

Our findings are the first to report that SCM-198 has considerable anti-neuroinflammatory effects on inhibiting microglial overactivation and might become a new potential drug candidate for AD therapy in the future.

## Introduction

Alzheimer’s disease (AD), the most common form of dementia among the elderly, is a chronic progressive disease characterized by cerebral deposition of senile plaques composed of amyloid-β (Aβ) peptides, intraneuronal neurofibrillary tangles originating from hyperphosphorylation of tau protein, profound loss of neurons and neuroinflammation [1–3]. Since the first patient with dementia described by Alois Alzheimer in 1907, many therapeutic strategies for AD have been proposed: acetylcholinesterase inhibitors, N-methyl-D-aspartate receptor antagonists, anti-amyloid therapies, drugs targeting tau protein and mitochondrial dysfunction, and so on [4]. Previous studies show that long-term use of NSAIDs lowers the risk of developing AD, alleviates neuroinflammation, suppresses senile plaques and improves tau pathology and cognition of different transgenic mice, but is accompanied by gastrointestinal, cardiovascular or nephrotoxicity [5,6].

Mounting evidence shows that inflammation plays a crucial role in AD progression. Microglia, primary immune cells of the brain, contribute largely to the neuroinflammatory responses. Under normal conditions, microglia take on a resting state with a ramified morphology and execute their surveillance and protective functions by extraction and retraction of their processes [7–9]. When the homeostasis of the central nervous system (CNS) is perturbed, they become activated with an amoeboid morphology accompanied by generations of free radicals, cytokines, chemokines and acute-phase proteins [10]. It is reported that Aβ aggregates and relative products from dead cells could activate microglia via Toll-like receptors (TLR) and receptors for advanced glycation end product-dependent pathways and promote release of proinflammatory factors, such as TNF-α, IL-1β and IL-6, which might in turn aggravate the disease [11]. In *postmortem* brains from AD patients and animals, most reactive microglia are located around dense-core Aβ plaques and elevated proinflammatory factors are also found in those brains which reveal the negative impact of neuroinflammation on AD progression [8]. Therefore, therapeutic drugs based on inhibiting microglial overactivation with less toxicity seem to be promising.

SCM-198 (4-guanidino-n-butyl syringate, also named Leonurine), a unique single compound existing only in *Herbaleonuri*, has been previously found to improve antioxidant capacity of myocardium [12], promote angiogenesis in ischemic myocardium and ameliorate endothelial dysfunction caused by hyperlipidemia [13]. During 2010 to 2011, SCM-198 was surprisingly found to be effective in stroke and Parkinson’s disease models via modulating mitochondrial functions and the redox state of the brain, respectively [14,15], which encouraged us to continuously explore its possible therapeutic potential in AD models.

Aβ peptides induce neurotoxicity in multiple ways, including oxidative stress, apoptosis or inflammation [16]. Meanwhile, SCM-198 has very good antioxidant, and anti-apoptotic neuro-and cardioprotective effects both *in vitro* and *in vivo*. Therefore, for investigating possible anti-neuroinflammatory mechanisms of SCM-198 in microglia, lipopolysaccharide (LPS), which is a very common agent for neuroinflammation studies, or aged Aβ_1-40_ peptides, was used to induce inflammatory responses *in vitro*. LPS, a component of Gram-negative bacterial cell wall, could activate TLR4 signalling, activate microglia and promote production of proinflammatory cytokines and related signaling pathways [17]. For *in vivo* studies, Aβ_1-40_-injected Sprague-Dawley (SD) rats were used to investigate the overall neuroprotective effect of SCM-198 on cognitive impairments and microglial overactivation. Our data indicated that SCM-198 could exert neuroprotective and anti-inflammatory effects both in Aβ_1-40_-injected rats and overactivated microglia, possibly via inhibition of NF-κB activation and c-Jun N-terminal kinase (JNK) pathways. This is also the first time that great hope could be placed on this new compound for its possible therapeutic potential in AD therapy in the near future.

## Methods

### Reagents

3-(4,5-dimethylthiazol-2-yl)-2, 5-diphenyltetrazolium bromide (MTT), BSA were purchased from Amresco (Solon, OH, USA). Ibuprofen (IBU), poly-d-lysine, phosphatase inhibitor cocktails, sulforhodamine B (SRB) and LPS were purchased from Sigma-Aldrich (St Louis, MO, USA). Inhibitors of mitogen-activated protein kinases (MAPKs) were from Cayman (Ann Arbor, MI, USA). Plasmocin was from Invivogen (San Diego, CA, USA). Primers were synthesized by Sangon (Shanghai, China) and all reagents for real-time reverse transcription-polymerase chain reaction (RT-PCR) and cell culture were from Takara (Dalian, China) and Gibco (Grand Island, NY, USA), respectively. Donepezil (DON) hydrochloride (purity > 99%) was supplied by Energy Chemical (Shanghai, China). SCM-198 (purity > 99%) was synthesized as previously described [15]. For *in vitro* studies, IBU, DON and SCM-198 were dissolved in dimethyl sulfoxide (DMSO) at concentrations of 0.5 M, 2 × 10^−2^ M and 10^−2^ M, respectively, and were diluted appropriately with cell culture medium (final DMSO concentration ≤ 1‰). For *in vivo* studies, DON and SCM-198 were dissolved in 0.9% sodium chloride solution containing 1% (w/v) sodium carboxymethylcellulose (CMC-Na). Lyophilized Aβ_1-40_ (ChinaPeptides, Shanghai, China) was first dissolved in sterilized distilled water followed by dilution with calcium-free PBS to a final concentration of 1 mg/ml. This solution was aggregated at 37°C for 7 days before its application in *in vitro* experiment or in the surgery.

### Cell culture

Cerebral cortex of newborn SD rats was separated and cut into small pieces after removing meninges and blood vessels to prepare mixed glial cells. Trypsinization (15 to 30 minutes, 37°C) with 0.125% trypsin was stopped with DMEM/F12 medium containing 10% FBS, 100 units/ml penicillin, 100 μg/ml streptomycin and 5 μg/ml plasmocin. The tissue was gently pipetted to obtain a single-cell suspension, which was then transferred to a new centrifuge tube after standing at room temperature (RT) for one to two minutes. This procedure was repeated three or four times. Then cells were centrifuged at 200 *g* for 5 minutes, resuspended in fresh DMEM/F12 medium and plated according to different protocols. Twenty-one days later, microglial cells were purified by mild trypsinization method [18]. For primary astrocyte culture, cortical mixed glial cells from SD rats were cultured for two weeks. When cells became confluent, astrocytes were purified by shaking at 350 rpm at 37°C for 12 hours. The purity (>95%) of primary microglia and astrocytes were confirmed with a mouse monoclonal CD11b antibody (1:200, Santa Cruz Biotechnology, Santa Cruz, CA,, USA) and a mouse monoclonal glial fibrillary acidic protein (GFAP) antibody (1:300, Cell Signaling, Danvers, MA,USA), respectively.

Cerebral cortex from fetuses of 17 to 18 days of gestation was used to prepare neurons, as described previously with minor modifications [19,20]. Preparation of single cell-suspension of neurons was the same with that of mixed glial cells. Cells were maintained in neurobasal medium supplemented with 2% B27, 0.5 mM L-glutamine, 100 units/ml penicillin and 100 μg/ml streptomycin. Medium was changed 24 hours after plating and every three days thereafter. Neurons cultured for 10 to 14 days were used in the experiments. The purity of neurons (>95%) was confirmed using a rabbit polyclonal MAP2 antibody (1:150, Cell Signaling, Danvers, MA, USA).

Immortalized murine BV-2 microglial cell line was first generated by Blasi*et al.* and retains many morphological and functional properties of primary microglia [21]. Cells were maintained in DMEM supplemented with 10% FBS, 100 units/ml penicillin and 100 μg/ml streptomycin, and were passed twice a week.

### Microglia/neuron co-culture

Microglia/neuron co-culture assay was performed as according to Yuekui Li *et al.* [22] with minor modifications. Neurons and BV-2 cells were separately seeded into 24-well (2 × 10^5^ neurons/well and 1 × 10^5^ BV-2 cells/insert) or 6-well (6 × 10^5^ neurons/well and 5 × 10^5^ BV-2 cells/insert) format transwell plates (0.4-μm pore-size, Corning Costar, St Louis, MO, USA). BV-2 cells were pretreated with or without 0.1 to 10 μM SCM-198 or 100 μM IBU for 2 hours and were stimulated with 1 μg/ml LPS for another 2 hours. Inserts containing BV-2 cells were then washed with fresh serum-free DMEM medium before placement into wells containing neurons to exclude the possible influence of residual LPS, SCM-198 or IBU. Twenty-four hours after co-culture, neuronal proteins were collected for Western blot analysis and neuronal viability was measured by MTT assay.

### Real-time RT-PCR analysis

Total RNA was extracted from BV-2 cells using TRIzol reagent (Takara, Dalian, China) according to the manufacturer’s instructions. One microgram of total RNA of each sample was reverse transcribed into cDNAs using the PrimeScript® RT Master Mix Perfect Real Time kit (Takara, Dalian, China). The resulting cDNAs were amplified by using a SYBR® Premix Ex TaqTM kit in iQ™5 real-time PCR detection system (Bio-Rad, Hercules, CA, USA) at 95°C for 30 seconds, 40 cycles at 94°C for 10 seconds and 60°C for 30 seconds, followed by 1 minute at 95°C, 1 minute at 60°C and finally 71 cycles at 60°C. Gene expressions of TNF-α, IL-1β, IL-6 and inducible nitric oxide synthase (iNOS) were analyzed with β-actin as an internal control. The primer sequences are listed below:

1. IL-6 [GenBank;NM_031168]: 5′-TTCCATCCAGTTGCCTTCTTG-3′ and 5′-TATCCTCTGTGAAGTCTCCTCTC-3′
2. IL-1β [GenBank:NM_008361]: 5′-ATCTCGCAGCAGCACATCAAC-3′ and 5′-TGTTCATCTCGGAGCCTGTAGT-3′
3. TNF-α [GenBank:NM_013693]: 5′-CGTGGAACTGGCAGAAGAGG-3′ and 5′-TCAGTAGACAGAAGAGCGTGGT-3′
4. iNOS [GenBank:NM_010927]: 5′-GGACGAGACGGATAGGCAGAGATT-3′ and 5′-AAGCCACTGACACTTCGCACAA-3′
5. β-actin [GenBank:NM_007393]: 5′-CTATTGGCAACGAGCGGTTCC-3′ and 5′-CAGCACTGTGTTGGCATAGAGG-3′

### Western blot analysis

Cells or rat hippocampus were lysed for 30 minutes on ice in radioimmunoprecipitation assay (RIPA)lysis buffer (Beyotime, Shanghai, China) supplemented with 1 mM phenyl methanesulfonyl fluoride, 1% phosphatase inhibitor cocktail 2 and 3. Supernatants were collected after centrifugation at 16,200 *g* for 20 minutes at 4°C and protein concentrations were measured using a BCA-100 Protein Quantitative Analysis kit (Shenergy Biocolor, Shanghai, China). For the analysis of NF-κB p65 translocation, nuclear proteins of BV-2 cells were extracted using NE-PER Nuclear and Cytoplasmic Extraction Reagents (Pierce, Rockford, IL, USA) according to the manufacturer’s instructions. Equal amounts of proteins were separated by 10 to 12% SDS-polyacrylamide gels and transferred onto polyvinylidene fluoride membranes (Millipore, Temecula, CA, USA). After blocking with 5% skim milk at RT for 1 hour, membranes were incubated with: polyclonal rabbit anti-NF-кB p65, monoclonal rabbit anti-histone H3 (D1H2), monoclonal mouse anti-IкBα (L35A5), monoclonal rabbit anti-phospho-p44/42 MAPK (ERK1/2) (Thr202/Tyr204), monoclonal rabbit anti-p44/42 MAPK (Erk1/2) (137 F5), monoclonal rabbit anti-phospho-p38 MAPK (Thr180/Tyr182) (D3F9), polyclonal rabbit anti-p38 MAPK, monoclonal rabbit anti-phospho-SAPK/JNK (Thr183/Tyr185) (81E11), monoclonal rabbit anti-SAPK/JNK (56G8), monoclonal mouse anti-phospho-Tau (Ser396) (PHF13), monoclonal mouse anti-Tau (Tau46), monoclonal rabbit anti-synaptophysin (D35E4), monoclonal rabbit anti-β-tubulin (1:1,000, Cell Signaling, Danvers, MA, USA), polyclonal rabbit anti-phospho-Tau (Thr205), polyclonal rabbit anti-phospho-Tau (Ser404) (1:1,000, Bioworld Technology, St Louis Park, MN, USA) primary antibodies overnight at 4°C, followed by incubation with appropriate horseradish peroxidase-conjugated secondary antibodies for 1 hour at RT. Blots were visualized using SuperSignal West Dura chemiluminescent substrate (Pierce, Rockford, IL, USA) in Alpha Imager Detection System (Alpha Innotech, San Leandro, CA, USA) and images were analyzed by ImageJ software (NIH, USA).

### Measurements of cell viability, cytokines, nitrite and LDH leakage

Microglial cells were preincubated with or without 0.1 to 10 μM SCM-198, IBU or MAPK inhibitors for 2 hours and stimulated with 1 μg/ml LPS for 24 hours or with 3 μM Aβ_1–40_ for 24 hours. Cell viability was measured by MTT assay according to an earlier protocol [15]. TNF-α and IL-1β levels in supernatant were measured using sandwich ELISA kits (Boatman Biotech, Shanghai, China) according to the manufacturer’s instructions. Nitrite was measured by Griess reaction: 100 μl of supernatants were mixed with 100 μl of Griess reagent (2% sulfanilamide, 5% H_3_P0_4_ and 0.2% N-(1-naphthyl) ethylenediaminedihydrochloride) and incubated for 15 minutes at RT. The absorbance was measured at 546 nm and NaNO_2_ (0 to 100 μM) was used as the standard. Morphological changes of primary microglia were observed using phase-contrast microscope (Carl Zeiss Inc., Thornwood, NY, USA) and quantified by radius ratio (radius ratio = maximum radius of cell/minimum radius of cell) using Image-Pro Plus 6.0 Analysis System (Media Cybernetics, Rockville, MD, USA). Primary cortical neurons were preincubated with or without 0.1 to 10 μM SCM-198 or DON for 2 hours and stimulated with 20 μM aged Aβ_1-40_ for 12 hours. Neuron viability was detected by SRB assay according to descriptions by Wai H Yu *et al.* [23] and lactate dehydrogenase (LDH) levels in the cell supernatants were determined using a commercial kit (Jiancheng Bioengineering, Nanjing, China).

### NF-кB nuclear translocation assay

BV-2 cells and primary microglia were pretreated with or without 1 μM SCM-198, 100 μM IBU or 20 μM DON and stimulated with 1 μg/ml LPS or 3 μM Aβ_1-40_ for 30 minutes. Cells were fixed with 4% paraformaldehyde and blocked with 10% BSA for 1 hour at RT, then incubated with monoclonal rabbit NF-κB p65 (D14E12) antibody (1:50, Cell Signaling, Danvers, MA, USA) overnight at 4°C, followed by stained with Alexa Fluor 488-conjugated goat anti-rabbit IgG (1:200, Molecular Probes, Eugene, OR, USA) for 2 hours in dark at RT. The nuclear translocation of NF-кB p65 was captured using fluorescence or confocal microscope (Carl Zeiss Inc., Thornwood, NY, USA).

### Surgery and drug administration

Sixty male SD rats (8 to 10 weeks old, 200 to 250 g) (BiKai, Shanghai, China) were randomly divided into 6 groups: sham group, Aβ_1-40_ group (both treated with 0.9% saline containing 1% CMC-Na), Aβ_1-40_ + SCM-198 15, 30, 60 mg/kg groups, DON group (1.0 mg/kg, as positive control). Drugs were given by gavage seven days before surgery, followed by daily administration until the end of the behavioral tests. Animals were provided with *ad libitum* food and water, and housed 5 per cage in a specific pathogen-free environment with 12-hour light/dark cycle and constant temperature (22°C ± 1°C). Seven days after drug pretreatment, rats were anesthetized with 7% chloral hydrate and positioned in a stereotaxic frame (Stoelting Co., Wood Dale, IL, USA). Two micrograms per liter of aggregated Aβ_1-40_ (5 μl/side, for Aβ_1-40_, Aβ_1-40_ + SCM198 15, 30, 60 mg/kg and DON groups) or vehicle (5 μl/side, for sham group) was bilaterally injected into the hippocampus at a rate of 0.5 μl/minute (coordinates: AP = −3.0 mm; ML = ± 2.2 mm; DA = −2.8 mm). Twelve days after surgery, Morris water maze (MWM) was applied to evaluate the spatial memory of the animals.

For investigating whether SCM-198 could improve the effect of DON, which is at the moment a palliative drug used in clinical management of AD, 45 male SD rats (8 to 10 weeks old, 200 to 250 g) (BiKai, Shanghai, China) were randomly divided into 5 groups: sham group, Aβ_1-40_ group (both treated with 0.9% saline containing 1% CMC-Na), Aβ_1-40_ + SCM-198 60 mg/kg groups, Aβ_1-40_ + DON 1 mg/kg group and Aβ_1-40_ + SCM-198 60 mg/kg + 1 mg/kg group DON group. Fifty days after surgery, MWM was applied to evaluate the possible long-lasting effect of SCM-198 and co-administration of SCM-198 and DON. All animal experiments conformed to guidelines of Regulations of Experimental Animal Administration of PR China (14 November 1988) and were approved by the Animal Ethics Committee of Fudan University.

### Morris water maze (MWM)

Animals were tested in the MWM for assessment of spatial reference memory in a room with constant temperature (23 ± 1°C) and humidity (around 50%). The apparatus consists of a transparent platform (9 cm in diameter) and a black Plexiglas circular pool (160 cm in diameter) which is equally divided into 4 quadrants. Spatial cues of different geometric shapes and colors were fixed on the upper wall of the pool and water temperature was kept at 21 ± 1°C. A four-day protocol was conducted: during the acquisition phase (from the first day to the third day), the platform was fixed in the middle of one quadrant (target quadrant) and submerged 1 to 2 cm below the water surface. Each rat was trained twice per day to find the invisible platform within one or two minutes randomly from two different starting points equidistant from the platform. The time for finding the platform was defined as escape latency. If animals failed to locate the hidden platform within two minutes, they were gently guided onto the platform and left there for ten seconds. On the fourth day, the platform was removed and each rat was given one minute to swim in the pool. Time spent in the target quadrant, swimming speed and paths was recorded and analyzed by the Doctor Mice software (Mobile Datum, Shanghai, China) [24].

### Histology

Rats were transcardially perfused with 0.9% saline followed by ice-cold 4% paraformaldehyde. The brains were fixed in the same fixative for 3 days and then in 30% sucrose for 4 days at 4°C. Thirty-micron coronal sections were made using a cryostat microtome (Thermo Fisher Scientific, Waltham, MA, USA). Sections were incubated in 0.3% Triton X-100 for 30 minutes, followed by 17 minutes in methanol/3% H_2_O_2_ (v/v, 1:1) solution. After blocked with a solution containing 5% BSA, 5% goat serum and 0.1% NaN_3_, sections were incubated with goat polyclonal anti-iba-1 antibody (1:500, Abcam, Cambridge, MA, USA) overnight at 4°C, then stained with MaxVision™ HRP-Polymer anti-Goat IHC Kit (Maixin-Bio, Fuzhou, China) for 15 minutes at RT. Microglia were visualized using a diaminobenzidine kit (Boster, Wuhan, China), and photographed by a phase-contrast microscope (Carl Zeiss Inc., Thornwood, NY, USA). Integrated optical density (IOD) of iba-1 expression was calculated using Image-Pro Plus 6.0 Analysis System (Media Cybernetics, Rockville, MD, USA).

### Statistical analysis

Data were expressed as mean ± SEM and analyzed either by one-way analysis of variance (ANOVA) or two-way repeated-measures ANOVA followed by Tukey’s *post-hoc* analysis using GraphPad Prism (La Jolla, CA, USA). Values of *P* < 0.05 were considered statistically significant.

## Results

### SCM-198 inhibited LPS- or Aβ_1-40_-induced proinflammatory mediator release in microglia and astrocytes and prevented morphological alterations in microglia

**Figure 1:**
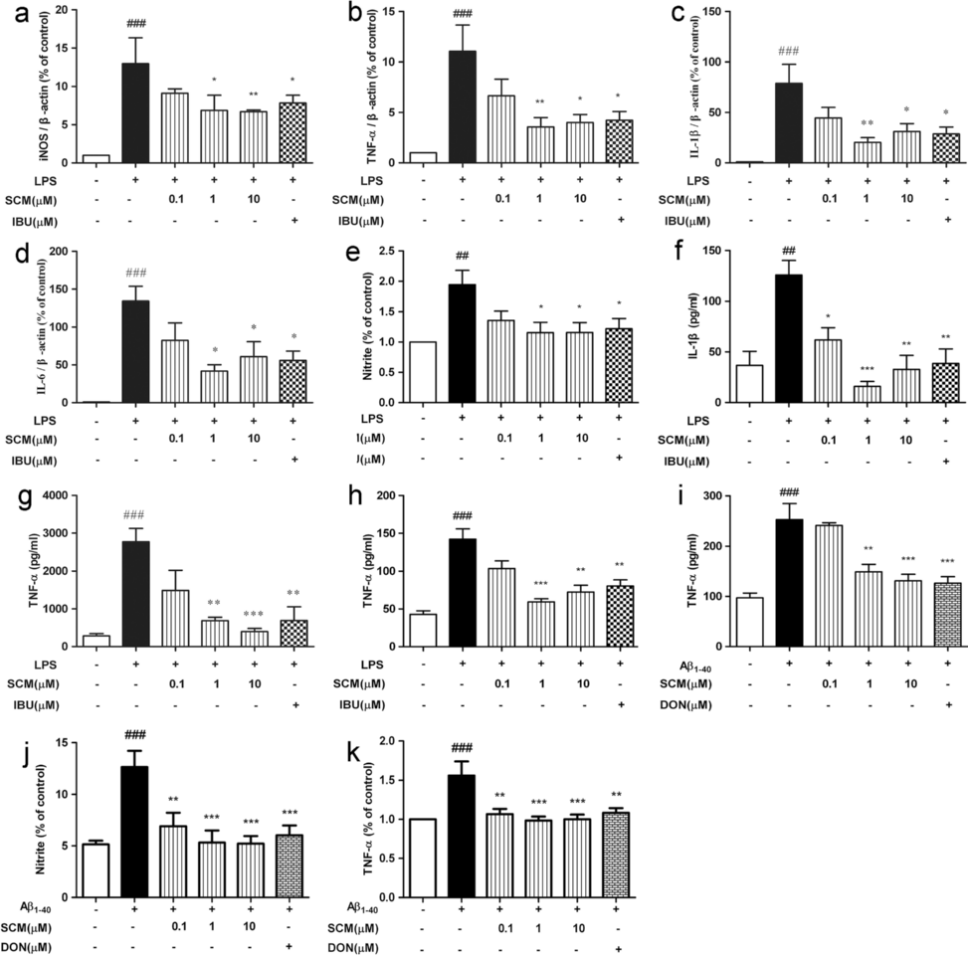
**SCM-198 inhibited lipopolysaccharide (LPS)- or Aβ**_**1-40**_ **-induced proinflammatory mediator release in microglia and astrocytes.** BV-2 cells were pretreated with indicated concentrations of SCM-198(SCM) or ibuprofen (IBU) for 2 hours, then stimulated with 1 μg/ml LPS for another 2 hours. mRNA expressions of iNOS **(a)**, TNF-α **(b)**, IL-1β **(c)** and IL-6 **(d)** were analyzed by real-time RT-PCR. BV-2 cells were pretreated with indicated concentrations of SCM or IBU for 2 hours, followed by 24-hour incubation with 1 μg/ml LPS, nitric oxide (NO) **(e)**, IL-1β **(f)**, TNF-α **(g)** levels in the cell culture medium were detected by Griess reaction and ELISA assay, respectively. Primary microglia were pretreated the same way as described above, followed by 24-hour incubation with 1 μg/ml LPS. TNF-α **(h)** level in the cell culture medium was measured by ELISA assay. BV-2 cells were pretreated with the indicated concentrations of SCM or 20 μM donepezil (DON) for 2 hours, followed by 24-hour stimulation with 3 μM Aβ_1-40_. TNF-α **(i)** in the cell culture medium was measured by ELISA assay. Primary astrocytes were pretreated with indicated concentrations of SCM or 20 μM donepezil (DON) for 2 hours, followed by 48-hour stimulation with 10 μM Aβ_1-40_. NO **(j)** and TNF-α **(k)** levels in the cell culture medium was measured by Griess reaction and ELISA assay, respectively. *P < 0.05, **P < 0.01, ***P < 0.001, Tukey’s test versus only LPS- or only Aβ_1-40_-treated group; #*P <* 0.05, ##*P <* 0.01, ###*P <* 0.001, Tukey’s test versus control group.

No obvious cytotoxicity was observed for 0.001 to 100 μM SCM-198 (data not shown). Possible anti-neuroinflammatory mechanisms of SCM-198 were studied mainly using BV-2 cells *in vitro*. As nitric oxide (NO) and cytokines, such as TNF-α, IL-1β and IL-6, are indicators of inflammatory process, we first investigated the inhibitory effects of SCM-198 on NO and proinflammatory cytokine release induced by LPS or Aβ_1-40_ in microglia. Two-hour pretreatment of BV-2 cells with 1 to 10 μM SCM-198 or 100 μM IBU could significantly suppress upregulation of iNOS, TNF-α, IL-1β and IL-6 mRNA expressions (*F *(5, 26) = 17.42, *P <* 0.0001, Figure 1a; *F *(5, 45) = 6.42, *P =* 0.0001, Figure 1b; *F *(5, 24) = 6.56, *P =* 0.0006, Figure 1c; *F *(5, 16) = 10.27, *P =* 0.0002, Figure 1d, respectively) induced by 2-hour exposure to 1 μg/ml LPS. Two-hour pretreatment of BV-2 cells with 1 to 10 μM SCM-198 or 100 μM IBU also inhibited NO, IL-1β and TNF-α productions after 24-hour incubation with 1 μg/ml LPS (*F *(5, 54) = 4.08, *P =* 0.0033, Figure 1e; *F*(5, 12) = 9.50, *P =* 0.0007, Figure 1f; *F*(5, 17) = 10.23, *P =* 0.0001, Figure 1g, respectively). TNF-α production induced by 24-hour exposure with 1 μg/ml LPS also decreased under pretreatment of 1 to 10 μM SCM-198 or IBU in primary microglia (*F *(5, 12) = 15.59, *P <* 0.0001, Figure 1h). Twenty-four-hour incubation with 3 μM Aβ_1-40_ doubled the production of TNF-α in BV-2 cells, which was effectively inhibited by 2-hour pretreatment of 1 to 10 μM SCM-198 or 20 μM DON (*F*(5, 18) = 14.74, *P <* 0.0001, Figure 1i). Forty-eight-hour stimulation of astrocytes with 10 μM Aβ_1-40_ also increased NO and TNF-α productions, which could also be significantly inhibited by 0.1 to 10 μM SCM-198 or 20 μM DON (*F*(5, 84) = 7.022, *P <* 0.0001, Figure 1j; *F *(5, 48) = 6.177, *P =* 0.0002, Figure 1k, respectively).

**Figure 2:**
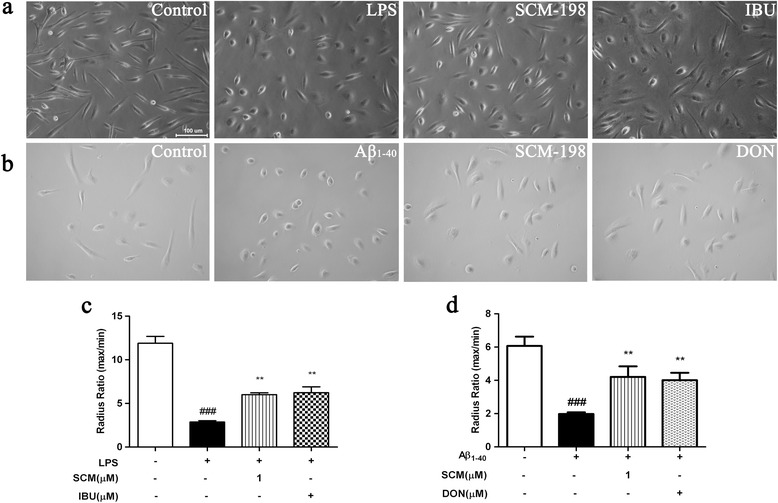
**SCM-198 prevented lipopolysaccharide (LPS)- or Aβ**_**1-40**_ **-induced morphological alterations in microglia.** BV-2 cells or primary microglia were pretreated with indicated concentrations of 1 μM SCM-198(SCM), 100 μM ibuprofen (IBU) or 20 μM donepezil (DON) for 2 hours, then stimulated with 1 μg/ml LPS **(a)** or 3 μM Aβ_1-40_ **(b)** for 24 hours. Morphological changes were quantified by measuring radius ratio (radius ratio = maximum radius/minimum radius) **(c-d)** (scale bar, 100 μm). Data represent mean ± SEM of more than three independent experiments. *P < 0.05, **P < 0.01, ***P < 0.001, Tukey’s test versus only LPS- or only Aβ_1-40_-treated group; #*P <* 0.05, ##*P <* 0.01, ###*P <* 0.001, Tukey’s test versus control group.

Morphological studies (Figure 2) showed that primary microglia became activated and took on an amoeboid shape (radius ratio decreased by nearly 75% and 67%, respectively) after 24-hour LPS or Aβ_1-40_ stimulation, while pretreatment of 1 μM SCM-198 or IBU or DON in some extent helped to prevent this cellular transformation (*F* (3, 12) = 48.66, *P <* 0.0001, Figure 2c; *F* (3, 336) = 9.794, *P <* 0.0001, Figure 2d).

### SCM-198 inhibited activation of JNK and NF-кB pathways induced by LPS in BV-2 cells

**Figure 3:**
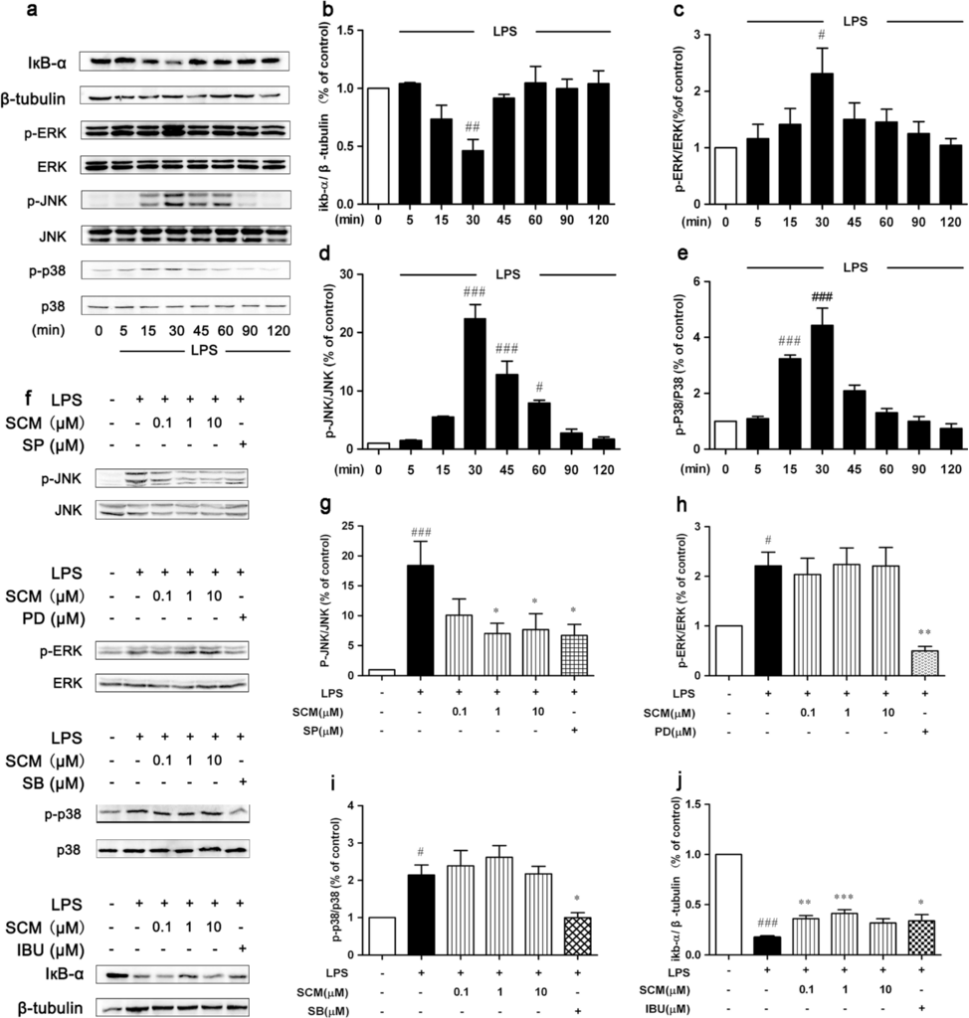
**SCM-198 inhibited lipopolysaccharide(LPS)-induced c-Jun N-terminal kinase (JNK) phosphorylation and IкB-α degradation in BV-2 cells.** Exposure of BV-2 cells to 1 μg/ml LPS led to a time-dependent IкB-α degradation **(a, b)** and phosphorylation of ERK (p-ERK) **(a, c)**, JNK (p-JNK) **(a, d)** and p38 (p-p38) **(a, e)**. BV-2 cells were pretreated with 0.1 to 10 μM SCM, 100 μM ibuprofen (IBU), 10 μM SP600125 (SP), 10 μM PD98059 (PD), or 20 μM SB203580 (SB) for 2 hours**,** followed by 30-minute LPS stimulation. SCM could inhibit JNK activation **(f, g)** and IкB-α degradation **(f, j)**, but not ERK **(f, h)** and p38 activation **(f, i)**. Data represent mean ± SEM of more than three independent experiments. *P < 0.05, **P < 0.01, ***P < 0.001, Tukey’s test versus only LPS-treated group; #*P* < 0.05, ##*P* < 0.01, ###*P* < 0.001, Tukey’s test versus control group.

**Figure 4:**
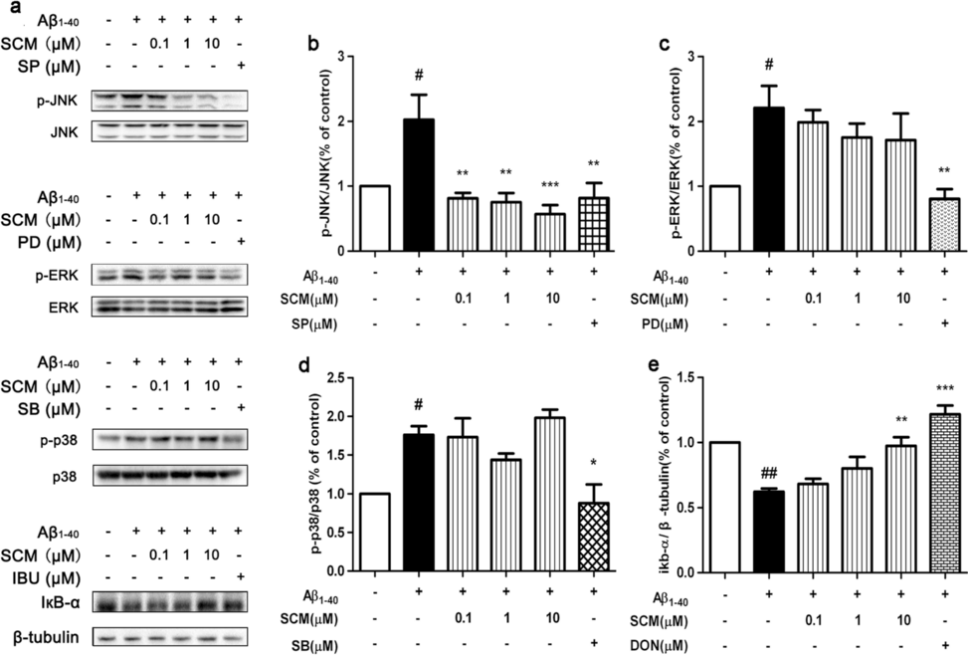
**SCM-198 also inhibited Aβ**_**1-40**_ **-induced c-Jun N-terminal kinase (JNK) phosphorylation and IкB-α degradation in BV-2 cells.** BV-2 cells were pretreated with 0.1 to 10 μM SCM, 20 μM donepezil (DON), 10 μM SP600125 (SP), 10 μM PD98059 (PD), or 20 μM SB203580 (SB) for 2 hours**,** followed by 30-minute Aβ_1-40_ stimulation. SCM could inhibit JNK activation **(a, b)** and IкB-α degradation **(a, e)**, but not ERK **(a, c)** and p38 activation **(a, d)**. Data represent mean ± SEM of more than three independent experiments. *P < 0.05, **P < 0.01, ***P < 0.001, Tukey’s test versus only Aβ_1-40_-treated group; #*P* < 0.05, ##*P* < 0.01, ###*P* < 0.001, Tukey’s test versus control group.

**Figure 5:**
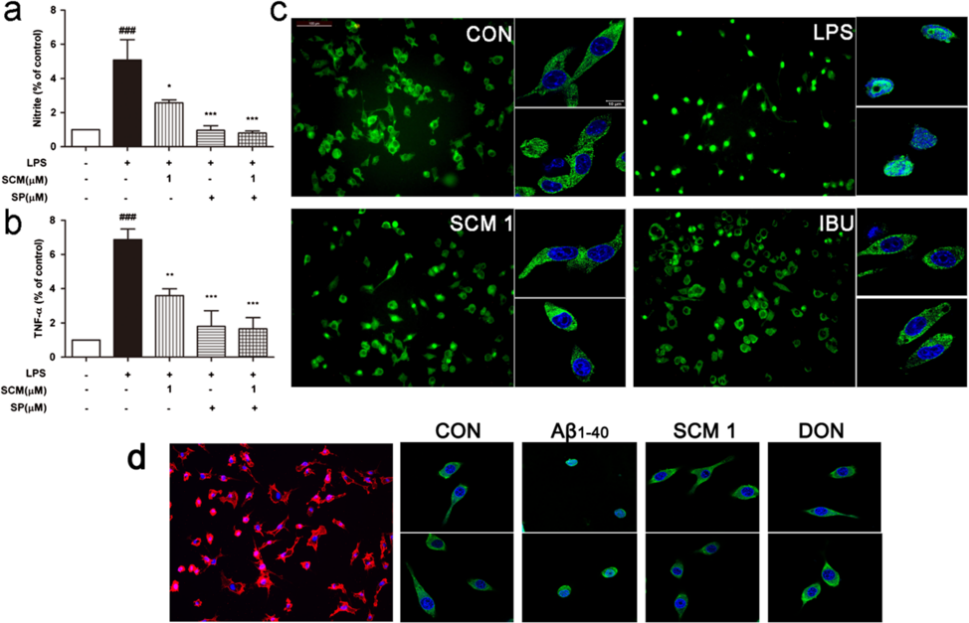
**SCM-198 inhibited microglia via c-Jun N-terminal kinase (JNK) and NF-кB pathways.** BV-2 cells were pretreated with 1 μM SCM or 10 μM SP (a specific inhibitor of JNK) for 2 hours, followed by 24-hour incubation with 1 μg/ml LPS. Nitric oxide (NO) **(a)** and TNF-α **(b)** in the cell culture medium were measured by Griess reaction and ELISA assay, respectively. The inhibitory effect of SCM on NO and TNF-α production could be mimicked by SP. BV-2 cells and primary microglia were both pretreated with 1 μM SCM, 100 μM ibuprofen (IBU) or 20 μM donepezil (DON) for 2 hours, and stimulated with 1 μg/ml lipopolysaccharide (LPS) **(c)** or Aβ_1-40_ **(d)** for 30 minutes; NF-кB p65 translocation was observed using fluorescence microscope (c, left panel of each group, BV-2 cells, scale bar, 100 μm) or confocal microscope (c-d, right panel of each group, primary microglia, scale bar, 10 μm). Primary microglia were stained using CD11b antibody (d, microglial marker, red) and 4',6-diamidino-2-phenylindole (DAPI) (nuclear staining, blue), purity of primary microglia is more than 95%. Data represent mean ± SEM of more than three independent experiments. *P < 0.05, **P < 0.01, ***P < 0.001, Tukey’s test versus only LPS-treated group; #*P* < 0.05, ##*P* < 0.01, ###*P* < 0.001, Tukey’s test versus control group.

One microgram per milliliter LPS induced inhibitor of NF-κB (IкB-α) degradation and phosphorylation of MAPKs (Figure 3a), including extracellular signal-regulated kinase (ERK), JNK and p38, in a time-dependent manner in BV-2 cells (*F* (7, 24) = 5.36, *P* = 0.0009, Figure 3b; *F* (7, 40) = 2.52, *P* = 0.0305, Figure 3c; *F* (7, 16) = 36.58, *P* < 0.0001, Figure 3d; *F* (7, 16) = 26.17, *P* < 0.0001, Figure 3e, respectively), while 3 μM Aβ_1-40_ could also mildly induce similar IкB-α degradation and MAPKs phosphorylation in BV-2 cells, and 30 minutes was chosen as the optimal time for LPS or Aβ_1-40_ stimulation. Two-hour pretreatment with SCM-198 could significantly inhibit JNK phosphorylation and IкB-α degradation, but not ERK and p38 (Figure 3f; *F* (5, 23) = 5.47, *P* = 0.0018, Figure 3g; *F* (5, 44) = 6.27, *P* = 0.0002, Figure 3h; *F* (5, 24) = 7.63, *P* = 0.0002, Figure 3i; *F* (5, 27) = 74.44, *P* < 0.0001, Figure 3j, respectively; Figure 4a; *F* (5, 30) = 6.585, *P* = 0.0003, Figure 4b; *F* (5, 24) = 4.772, *P* = 0.0036, Figure 4c; *F* (5, 18) = 7.959, *P* = 0.0004, Figure 4d; *F* (5, 18) = 16.00, *P* < 0.0001, Figure 4e, respectively). Inhibitory effects of SCM-198 on NO and TNF-α production could be mimicked by 10 μM SP600125, a specific inhibitor of JNK, in BV-2 cells (*F* (4, 24) = 10.42, *P* < 0.0001, Figure 5a; *F* (4, 53) = 16.55, *P* < 0.0001, Figure 5b, respectively). NF-кB, ubiquitously expressed in almost every organ, plays crucial roles in inflammation and was found to be activated around senile plaques in AD patients’ brains [25]. In our study, a 30-minute stimulation of 1 μg/ml LPS or 3 μM Aβ_1-40_ activated the NF-кB signalling pathway and induced p65 translocation into the nucleus in both BV-2 cells (Figure 5c, left panel of each group) and primary microglia (Figure 5c, right panel of each group, Figure 5d). Two-hour pretreatment with 1 μM SCM-198 or 100 μM IBU or 20 μM DON could significantly diminish this effect (Figure 5c-5d).

### SCM-198 directly protected neurons or indirectly protected neurons via modulating microglia in microglia/neuron co-culture system

**Figure 6:**
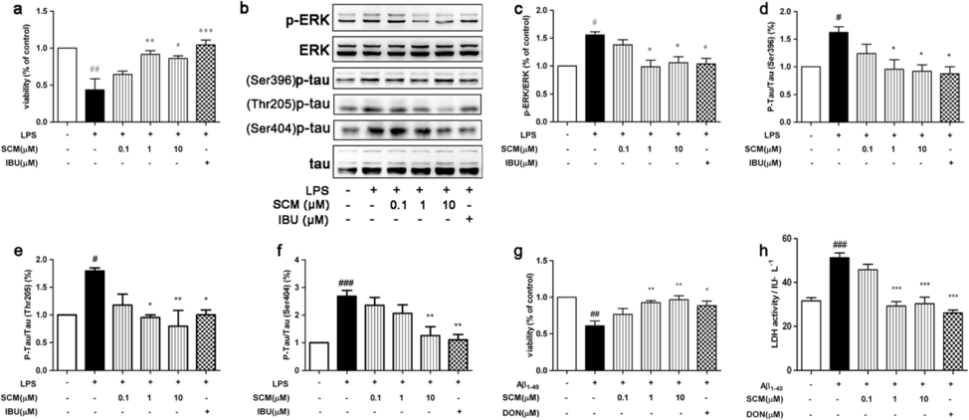
**SCM-198 could directly protect neurons from Aβ**_**1-40**_ **toxicity or indirectly protect neurons via modulating microglia in microglia/neuron co-culture system.** BV-2 cells were pretreated with 0.1 to 10 μM SCM or 100 μM ibuprofen (IBU), and stimulated with 1 μg/ml lipopolysaccharide (LPS) for 2 hours, washed with fresh DMEM medium and co-cultured with cortical neurons for 24 hours. Neuronal viability was measured by 5-diphenyltetrazolium bromide (MTT) assay **(a)** and phosphorylation of ERK (p-ERK) and tau (p-tau) of neurons were detected by Western blot **(b-f)**. Primary cortical neurons were pretreated with indicated concentrations of SCM or 20 μM donepezil (DON) for 2 hours, followed by 12-hour stimulation of 20 μM Aβ_1-40_. Neuronal viability **(g)** and LDH leakage **(h)** were measure by sulforhodamine B (SRB) assay and the lactate dehydrogenase (LDH) kit. Data represent mean ± SEM of more than three independent experiments. *P < 0.05, **P < 0.01, Tukey’s test versus only LPS- or only Aβ_1-40_-treated group; #*P* < 0.05, ##*P* < 0.01, Tukey’s test versus control group.

As SCM-198 could effectively inhibit microglial overactivation, we then ventured into how these ‘SCM-198- or IBU-pretreated’ microglia would interact with neurons. A co-culture system was applied to investigate whether SCM-198 could protect neurons indirectly via directly suppressing overactivated microglia. LPS-preactivated or ‘SCM-198- or IBU-pretreated’ BV-2 cells in inserts were washed twice with fresh DMEM medium to remove residual LPS, SCM-198 or IBU. These washed cells in inserts were then placed into wells containing primary neurons and were co-cultured for 24 hours. Between inserts and wells, there is a semi-permeable membrane (0.4-μm pore-size) which blocks the direct contact between neurons and microglia, but allows exchange of molecules. LPS-preactivated BV-2 cells caused a decrease in neuronal viability which was reversed by ‘SCM-198- or IBU-pretreated’ microglia and 1 μM SCM-198 turned out to be the optimal dose (*F* (5, 12) = 9.984, *P* = 0.0006, Figure 6a). Accordingly, Western blot showed that phosphorylation of ERK and tau in neurons were repressed by SCM-198 (Figure 6b, *F* (5, 50) = 4.27, *P* = 0.0026, Figure 6c; *F* (5, 30) = 3.40, *P* = 0.0150, Figure 6d, *F* (5, 12) = 5.599, *P* = 0.0069, Figure 6e; *F* (5, 24) = 8.544, *P* < 0.0001, Figure 6f, respectively), indicating that SCM-198 could indirectly protect primary neurons through suppressing microglial overactivation. Meanwhile, SCM-198 could also directly protect neurons from 20 μM Aβ_1-40_-induced neuronal death (*F* (5, 18) = 7.07, *P =* 0.0008, Figure 6g) and LDH leakage (*F* (5, 30) = 23.41, *P <* 0.0001, Figure 6h).

### SCM-198 ameliorated cognitive deficits of Aβ_1-40_ injected SD rats in MWM test

**Figure 7:**
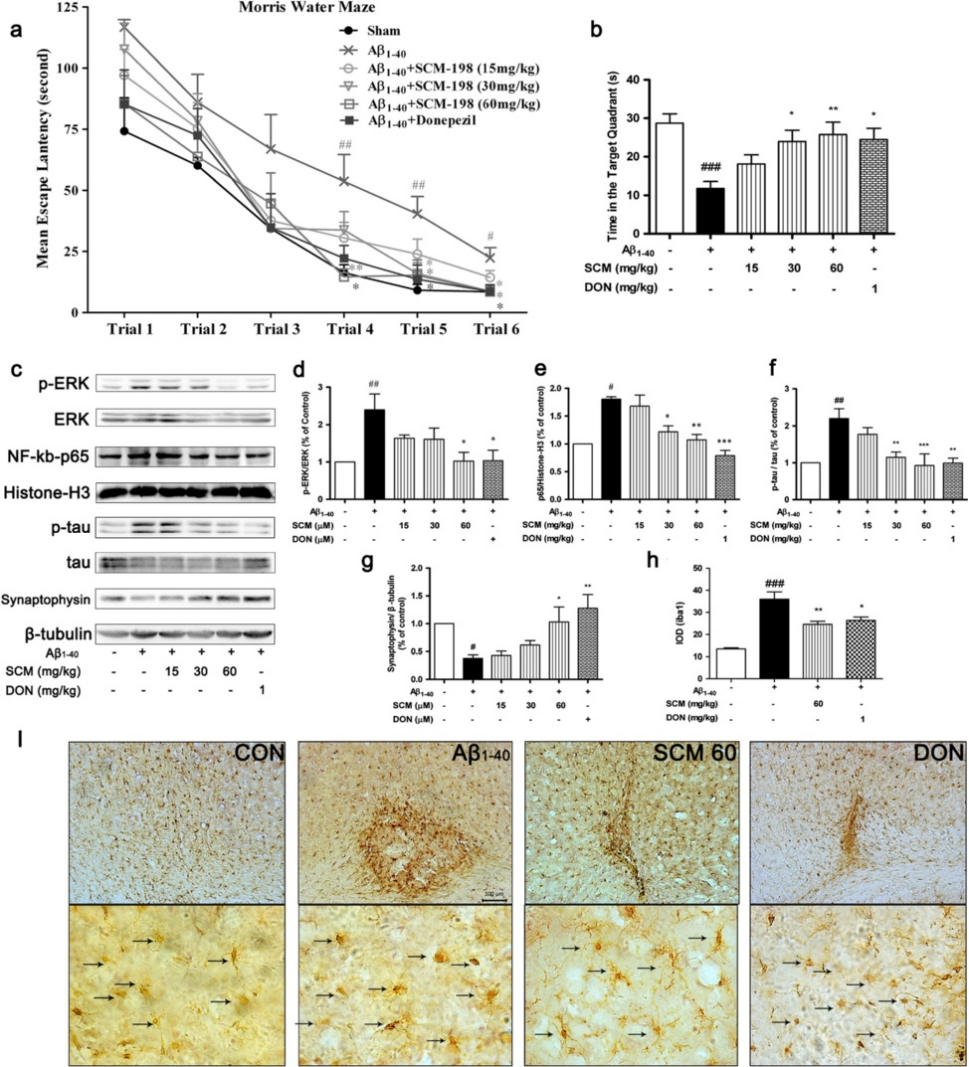
**SCM-198 improved cognitive performances of Aβ**_**1-40**_ **-injected Sprague-Dawley (SD) rats in the Morris water maze (MWM) test, alleviated microglial activation, suppressed phosphorylation of extracellular signal-regulated kinase (ERK) and tau, inhibited NF-кB p65 translocation and synaptophysin loss** ***in vivo*** **.** SD rats were subjected to bilateral microinjections of 2 μg/μl aggregated Aβ_1-40_ (5 μl/side) or vehicle into hippocampus and MWM test was conducted 12 days after surgery. **(a)** Mean escape latency from Day 1 to Day 3; **(b)** time spent in target quadrant. Data represent mean ± SEM of nine to ten rats per group. *P < 0.05, **P < 0.01, Tukey’s test versus only Aβ_1-40_-treated group; #*P* < 0.05, ##*P* < 0.01, ###*P* < 0.001, Tukey’s test versus sham group. Phosphorylation of ERK (p-ERK) **(c, d)**, nuclear p65 (NF-кB-p65) **(c, e)**, phosphorylation of tau (p-tau) **(c, f)** and synaptophysin loss **(c, g)** were analyzed by Western blot (n = 5 rats/group). **(i)** Examples of iba-1 staining for microglia (dark brown, indicated by arrows) in hippocampus (scale bar, 100 μm; n = 3 rats/group). **(h)** Integrated optical density (IOD) for of iba-1 staining for microglia. Data represent mean ± SEM. *P < 0.05, **P < 0.01, ***P < 0.001, Tukey’s test versus only Aβ_1-40_-treated group; #*P* < 0.05, ##*P* < 0.01, Tukey’s test versus sham group.

To further explore neuroprotective effects of SCM-198 *in vivo*, SD rats with bilateral intrahippocampal injections of aggregated Aβ_1-40_ were applied. As the experiment progressed, average escape latencies of all groups gradually decreased, with no significant differences observed from trial 1 to trail 3 (*F* (5, 53) = 2.06, *P* = 0.085; *F* (5, 53) *=* 0.98, *P* = 0.440; *F* (5, 53) = 1.11, *P* = 0.3668, respectively, Figure 7a). Up to trial 4, a dramatically significant decrease of escape latency was observed in 60 mg/kg SCM-198-treated group as compared with that of only Aβ_1-40_-treated group (*F* (5, 53) = 4.70, *P* = 0.0013, Figure 7a). In trial 5 and trial 6, rats administered with 30 mg/kg SCM-198 also showed considerable cognitive improvements (*F* (5, 53) = 4.10, *P* = 0.0032; *F* (5, 53) = 4.00, *P* = 0.0037, respectively, Figure 7a). Time spent in the target quadrant was assessed during probe trial (Day 4). Figure 7b showed that SCM-198 enhanced spatial memory of rats in a dose-dependent manner (*F* (5, 53) *=* 5.44, *P* = 0.0004, Figure 7b). Two-way repeated-measures ANOVA analysis showed a significant effect of drug treatment (*F* (5, 265) = 8.667, *P* < 0.0001) and trial effect (*F* (5, 265) = 84.80, *P* < 0.0001). Body weight remains normal and no statistical differences were found in swimming speed of rats between groups throughout the experiment (data not shown). DON (1 mg/kg), a first-line inhibitor of acetylcholinesterase and now clinically used for AD treatment, was used as the positive control [26]. Taken together, behavioral data indicated that SCM-198 could ameliorate cognitive impairments in a time- and dose-dependent manner with 60 mg/kg as the optimal dose.

### SCM-198 alleviated microglial activation, decreased phosphorylation of ERK and tau, inhibited synaptophysin loss and NF-кB p65 activation *in vivo*

Intrahippocampal injections of Aβ_1-40_ led to elevated ERK phosphorylation, NF-кB p65 activation, increased tau phosphorylation, and synaptophysin loss, which were significantly reversed by SCM-198 treatment in a dose-dependent manner, with 60 mg/kg as the optimal dose (Figure 7c, *F* (5,27) = 4.44, *P* = 0.0045, Figure 7d; *F* (5,18) = 13.23, *P* < 0.0001, Figure 7e; *F* (5,36) = 6.93, *P* = 0.0001, Figure 7f; *F* (5,31) = 6.13, *P* = 0.0005, Figure 7g, respectively). Immunostains of brain slices against iba-1 (Figure 7i) showed that Aβ_1-40_ injections induced excessive microglial activation at and around the injection site and SCM-198 at 60 mg/kg and DON could attenuate this activation (*F* (3, 20) = 22.04, *P* < 0.0001, Figure 7h).

### Synergistic effects of SCM-198 and donepezil on cognitive impairments in a chronic rat AD model induced by Aβ_1-40_

**Figure 8:**
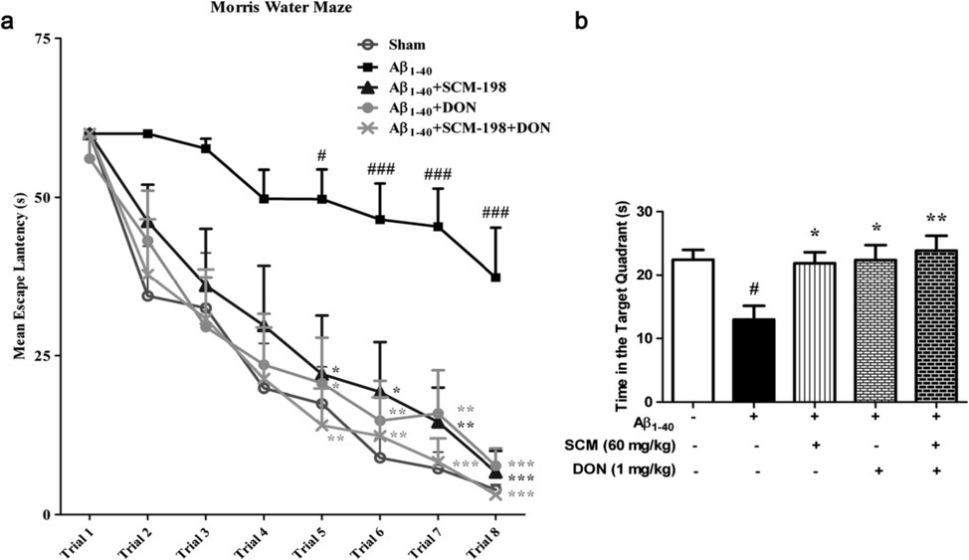
**Synergistic effects of SCM-198 and donepezil on cognitive impairments in a chronic rat Alzheimer’s disease (AD) model induced by Aβ**_**1-40**_ **.** SD rats were subjected to bilateral microinjections of 2 μg/μl aggregated Aβ_1-40_ (5 μl/side) or vehicle into hippocampus and Morris water maze (MWM) test was conducted 50 days after surgery. **(a)** Mean escape latency from Day 1 to Day 4; **(b)** time spent in target quadrant. Data represent mean ± SEM of nine rats per group. *P < 0.05, **P < 0.01, Tukey’s test versus only Aβ_1-40_-treated group; #*P* < 0.05, ##*P* < 0.01, ###*P* < 0.001, Tukey’s test versus sham group.

As described in the Material and Methods section, 45 male rats were pretreated with vehicle, 60 mg/kg SCM-198, 1 mg/kg DON or co-administrated with SCM-198 and DON for 7 days. Fifty days after surgery, rats of only the Aβ_1-40_-injected group showed more severe cognitive impairments in spatial reference memory as compared with that of rats of 12-day recovery from surgery. Even up to trial 8, rats of only the Aβ_1-40_-injected group still needed 37.3 seconds in average to find the invisible platform. No significant differences were observed from trial 1 to trail 4 (*F* (4, 40) = 1.292, *P* = 0.2895; *F* (4, 40) *=* 2.078, *P* = 0.1018; *F* (4, 40) = 2.40, *P* = 0.066; *F* (4, 40) = 2.603, *P* = 0.0502, respectively, Figure 8a). From trial 5 to trial 8, therapeutic effects of SCM-198, DON and co-administration of SCM and DON became statistically significant and animals of co-administration group showed the best performances. (*F* (4, 40) = 4.517, *P* = 0.0042; *F* (4, 40) *=* 6.299, *P* = 0.0005; *F* (4, 40) = 9.255, *P* < 0.0001; *F* (4, 40) = 12.75, *P* < 0.0001, respectively, Figure 8a). Two-way repeated-measures ANOVA analysis showed an extremely significant effect of drug treatment (*F* (4, 280) = 21.41, *P* < 0.0001) and trial effect (*F* (7, 280) = 35.76, *P* < 0.0001). Body weight remains normal and no statistical differences were found in swimming speed of rats between groups throughout the experiment (data not shown). Time spent in the target quadrant was also assessed during probe trial (Day 5). Figure 8b showed that 60 mg/kg SCM-198, 1 mg/kg DON and co-administration of SCM-198 and DON all lengthened their stay in target quadrant with rats of co-administration group spending the longest time (*F* (5, 53) *=* 4.562, *P* = 0.004, Figure 8b), indicating that SCM-198 could effectively improve the therapeutic effect of DON.

## Discussion

The role neuroinflammation plays in the pathological development of AD still remains controversial today, as inflammation itself is an innate defense against both endogenous and exogenous insults under normal physiological conditions. In neurodegenerative diseases like AD, chronic long-lasting inflammation is considered, to a great extent, as the driving force or even initiator of the disease [3]. During this process, ramified resting microglia undergo morphological changes including deramification, process shortening and thickening and finally development into its activated amoeboid form. Neurotoxic factors such as proinflammatory cytokines and chemokines are subsequently released from activated microglia and lead to neuronal damage [27].

For the indispensable role of microglia in the brain, therapeutic strategies of curbing microglial neurotoxicity without affecting its viability would be feasible. Extensive literatures have documented that IBU, one of the most commonly used NSAIDs, could significantly inhibit activation of human primary microglia or THP-1 macrophages [28] and suppress brain inflammation [5,29–31]. Thus, IBU was chosen as the positive control for *in vitro* study. Our data obtained from both primary microglia and BV-2 cell line indicated considerable inhibitory effects of SCM-198 on overactivated microglia via suppressing proinflammatory cytokines and NO productions. Possible underlying mechanisms were demonstrated to be, at least partially, through the inhibitions of NF-кB and JNK pathways. Microglial phenotype transition from amoeboid back to ramified morphology was observed after SCM-198 treatment (Figure 2), which was consistent with data from cytokine and NO assays in microglia. Co-culture and *in vivo* data provided further validations for the neuroprotective effects of SCM-198, which alleviated neuroinflammation via modulating microglia and therefore improved overall cognitive performances of rats. One thing of note is the optimal dose of SCM-198 in *in vitro* experiments. In most cases, SCM-198 at 1 μM exerted the best inhibitory effect in microglia, while 10 μM sometimes became the optimal dose. This could be possibly ascribed to different sensitivities between cell lineages (primary microglia versus BV-2 cells) (Figure 1g and 1h). Besides, 10 μM SCM-198 was more effective than 1 μM SCM-198 in inhibiting NO production (Figure 1a and 1e), while SCM-198 at 1 μM inhibited transcriptions of proinflammatory cytokines more effectively (Figure 1b-1d). We guessed that 10 μM SCM-198 not only inhibited transcriptions of cytokines, but also introduced some unknown mechanisms which unregulated NO production to some extent. On the other hand, SCM-198 at 1 and 10 μM could both inhibit proinflammatory factors, which means a relatively broad therapeutic window of this compound. Figure 1i showed that 3 μM Aβ_1-40_ also upregulated TNF-α release after 24-hour incubation and SCM-198 at 1 and 10 μM significantly inhibited this elevation. Meanwhile, in Figure 6g-6h, neurons died when directly treated with 20 μM Aβ_1-40_ for 12 hours and no neuronal loss was observed when they were treated with 3 μM Aβ_1-40_ for 24 hours (data not shown). This means that 3 μM Aβ_1-40_ is sublethal for primary neurons while it could induce significant elevation of TNF-α in microglia. Besides, astrocytes seemed less sensitive to Aβ_1-40_ than microglia, as up to 3 times higher concentration of Aβ_1-40_ and 48 hour stimulation increased astrocytic NO and TNF-α productionsas compared to 3 μM Aβ_1-40_ and 24 hour stimulation in microglia (Figure 1j-1k). In another words, microglial overactivation might play the key role in neurodegeneration especially when the concentration of Aβ_1-40_ remains low and sublethal for neurons. The differences of cognitive performances in MWM between rats of 12-day recovery and 50-day recovery could also be explained in this way: Aβ inoculation in hippocampus needs enough time to form plaques and affect neurons in the brain, so Aβ aggregates at low concentration would possibly activate microglia at the very beginning, then parenchymal Aβ aggregates would gradually affect neurons and result in more severe cognitive impairments overtime.

LPS is able to elicit inflammatory responses in monocytes and macrophages by activating multiple intracellular signaling pathways including NF-кB pathway and three MAPK pathways (ERK, JNK, and p38). Activated downstream transcription factors subsequently enter the nuclei, bind to the promoter regions and initiate transcriptions of various proinflammatory mediators such as NO, TNF-α, IL-1β and so on [32,33]. In our study, 1 μg/ml LPS indeed induced phosphorylation of MAPKs with the peak level at 30 minutes. However, inhibition of phosphorylation by SCM-198 in microglia could only be observed for JNK, but not for ERK and p38. A recent study in our lab found that SCM-198 protected against TNF-α-induced inflammation in human umbilical vein endothelial cells via inhibiting p38 activation, but not ERK and JNK [34]. This divergence could be ascribed to the different cell line and inflammatory initiator applied. It is considered that LPS and TNF-α elicit cellular inflammatory responses mainly through Toll-like and TNF receptors [35,36], respectively. Thus the two different conclusions above could be valid but need further investigations. Inhibitory effects of SCM-198 on JNK phosphorylation and TNF-α release can be mimicked by SP600125 (a JNK inhibitor), indicating that SCM-198 exerts neuroprotective effects, at least partially, via inhibiting both NF-кB and JNK pathways in microglia.

As Aβ deposits, neurofibrillary tangles and dystrophic neurites are widely accepted hallmarks of AD. We herein used pre-aggregated Aβ_1-40_ as the *in vivo* inflammation inducer. Many literatures have stated that intrahippocampal injections of Aβ_1-40_ or Aβ_1-42_ could activate glial cells, elicit neuroinflammation and cause cognitive impairments in rodents [37–39]. In our study, Aβ_1-40_ injections caused microglial activation, synaptophysin loss, elevated phosphorylation of tau, ERK and NF-кB p65, which were later significantly reversed by SCM-198. Our unpublished data obtained from HPLC analysis showed that SCM-198 administrated by gavage could be detected in rat brains, which could be considered as supportive evidence that SCM-198 could penetrate the blood-brain barrier and directly exert neuroprotective effects in CNS (Shi *et al*., unpublished data).

Interestingly, SCM-198 inhibited NF-кB and JNK pathways in activated microglia, while it inhibited ERK and NF-кB pathways in co-cultured neurons and rat hippocampus. Possible explanations for the difference were as follows: 1) in the co-culture paradigm, neurons were directly stimulated by molecules released from pretreated microglia, but not directly by LPS and SCM-198, which were removed from the media before microglia/neuron co-culture; 2) Other studies have proved that activated microglia upregulated p-ERK with no change in total ERK in neurons and rodents’ brains and this elevation of p-ERK was accompanied by neuronal dysfunctions and cognitive impairments of animals [40–42]; 3) Hence, elevation of p-ERK in co-cultured neurons and tissues was possibly an overall consequence of the interactions between neurons and LPS- or Aβ-activated microglia. Therefore, we concluded that SCM-198 could either directly protect neurons from Aβ_1-40_ toxicity or indirectly protect neurons against synaptophysin loss and elevations of p-tau, p-ERK and p-p65 of NF-кB via directly suppressing NF-кB and JNK pathways in activated microglia.

Further investigations will be necessary to clarify how SCM-198 interacts with neurons and astrocytes. Several other transgenic AD models will be needed to further verify neuroprotective effects or unravel new potential mechanisms of SCM-198. Taken together, our study, for the first time, demonstrated that SCM-198 possessed considerable anti-neuroinflammatory effect both *in vitro* and *in vivo* and therefore protected co-cultured neurons and improved overall cognitive performances of rats. Hence, our data may provide new insights into AD treatment with SCM-198 in the near future.

## Conclusions

In summary, this is the first time that SCM-198 was found to have considerable anti-inflammatory effects in microglia and in Aβ_1-40_-injected SD rats, indicating its potential as a drug candidate for AD treatment in the future. SCM-198 may directly inhibit overactivated microglia, maintain their ramified morphology, decrease proinflammatory cytokines via NF-кB and JNK pathways and therefore indirectly protect co-cultured neurons. Besides, when directly applied to neurons, SCM-198 decreased neuronal death and LDH leakage caused by Aβ_1-40_ stimulation. *In vivo* Aβ_1-40_ injection caused impairments of spatial memory and microglial overactivation, which were reversed by SCM-198 at 30 mg/kg and 60 mg/kg. In the chronic rat AD model, co-administration of SCM-198 and DON resulted in better cognitive performances of rats in the MWM test, indicating that SCM-198 could not only be used independently for AD treatment in the future, but that it could be used as an adjuvant to improve the therapeutic effect of DON. Further investigations will be necessary to clarify how SCM-198 interacts with neurons and astrocytes. Several other transgenic AD models will be needed to further verify neuroprotective effects or unravel new potential mechanisms of SCM-198.

## Abbreviations

Aβ: amyloid-β
AD: Alzheimer’s disease
ANOVA: analysis of variance
BSA: bovine serum albumin
CMC-Na: sodium carboxymethylcellulose
CNS: central nervous system
DAPI: 4',6-diamidino-2-phenylindole
DMEM: Dulbecco’s modified Eagle’s medium
DMSO: dimethyl sulfoxide
DON: donepezil
ELISA: enzyme-linked immunosorbent assay
ERK: extracellular signal-regulated kinase
FBS: fetal bovine serum
GFAP: glial fibrillary acidic protein
HPLC: high performance liquid chromatography
IBU: ibuprofen
Ig: immunoglobulin
IL: interleukin
IкB: inhibitor of NF-κB
iNOS: inducible nitric oxide synthase
IOD: integrated optical density
JNK: c-Jun N-terminal kinase
LDH: lactate dehydrogenase
LPS: lipopolysaccharide
MAPKs: mitogen-activated protein kinases
MTT: 5-diphenyltetrazolium bromide
MWM: Morris water maze
NF-κB: nuclear factor-κB
NO: nitric oxide
NSAIDs: nonsteroidal anti-inflammatory drugs
PBS: phosphate-buffered saline
RIPA: radioimmunoprecipitation assay
RT: room temperature
RT-PCR: real-time reverse transcription-polymerase chain reaction
SCM-198: 4-guanidino-n-butyl syringate
SD: Sprague-Dawley
SRB: sulforhodamine B
TLR: Toll-like receptors
TNF: tumor necrosis factor

## References

[1] LaFerla F, Green K, Oddo S (2007). Intracellular amyloid-beta in Alzheimer’s disease. Nat Rev Neurosci.

[2] Blasko I, Stampfer-Kountchev M, Robatscher P, Veerhuis R, Eikelenboom P, Grubeck-Loebenstein B (2004). How chronic inflammation can affect the brain and support the development of Alzheimer’s disease in old age: the role of microglia and astrocytes. Aging Cell.

[3] Wyss-Coray T (2006). Inflammation in Alzheimer disease: driving force, bystander or beneficial response?. Nat Med.

[4] Mangialasche F, Solomon A, Winblad B, Mecocci P, Kivipelto M (2010). Alzheimer’s disease: clinical trials and drug development. Lancet Neurol.

[5] Lim G, Yang F, Chu T, Chen P, Beech W, Teter B, Tran T, Ubeda O, Ashe K, Frautschy S, Cole G (2000). Ibuprofen suppresses plaque pathology and inflammation in a mouse model for Alzheimer’s disease. J Neurosci.

[6] Carreras I, Mckee A, Choi J, Aytan N, Kowall N, Jenkins B, Dedeoglu A (2013). R-flurbiprofen improves tau, but not A beta pathology in a triple transgenic model of Alzheimer’s disease. Brain Res.

[7] Mandrekar-Colucci S, Landreth G (2010). Microglia and inflammation in Alzheimer’s Disease. CNS Neurol Disord Drug Targets.

[8] Schlachetzki J, Hüll M (2009). Microglial activation in Alzheimer’s disease. Curr Alzheimer Res.

[9] Streit W, Xue Q (2009). Life and death of microglia. J NeuroimmunePharmacol.

[10] Cameron B, Landreth G (2010). Inflammation, microglia, and Alzheimer’s disease. Neurobiol Dis.

[11] Glass C, Saijo K, Winner B, Marchetto M, Gage F (2010). Mechanisms underlying inflammation in neurodegeneration. Cell.

[12] Liu X, Pan L, Chen P, Zhu Y (2010). Leonurine improves ischemia-induced myocardial injury through antioxidative activity. Phytomedicine.

[13] Liu X, Pan L, Yang H, Gong Q, Zhu Y (2012). Leonurine attenuates lipopolysaccharide-induced inflammatory responses in human endothelial cells: involvement of reactive oxygen species and NF-kappa B pathways. Eur J Pharmacol.

[14] Loh K, Qi J, Tan B, Liu X, Wei B, Zhu Y (2010). Leonurine protects middle cerebral artery occluded rats through antioxidant effect and regulation of mitochondrial function. Stroke.

[15] Shi X, Hong Z, Liu H, Zhang Y, Zhu Y (2011). Neuroprotective effects of SCM198 on 6-hydroxydopamine-induced behavioral deficit in rats and cytotoxicity in neuronal SH-SY5Y cells. NeurochemInt.

[16] Hardy J, Selkoe D (2002). Medicine - The amyloid hypothesis of Alzheimer’s disease: progress and problems on the road to therapeutics. Science.

[17] Zhang W, Qin L, Wang T, Wei S, Gao H, Liu J, Wilson B, Liu B, Zhang W, Kim H, Hong J: **3-Hydroxymorphinan is neurotrophic to dopaminergic neurons and is also neuroprotective against LPS-induced neurotoxicity.** *FASEB J* 2004, **18:**395−+. 10.1096/fj.04-1586fje 15596482

[18] Saura J, Tusell J, Serratosa J (2003). High-yield isolation of murine microglia by mild trypsinization. Glia.

[19] Brewer G, Torricelli J, Evege E, Price P (1993). Optimized survival of hippocampal-neurons in B27-supplemented neurobasal (TM), a new serum-free medium combination. J Neurosci Res.

[20] Li Y, Wang J, Sheng J, Liu L, Barger S, Jones R, Van Eldik L, Mrak R, Griffin S (1998). S100 beta increases levels of beta-amyloid precursor protein and its encoding mRNA in rat neuronal cultures. J Neurochem.

[21] Blasi E, Barluzzi R, Bocchini V, Mazzolla R, Bistoni F (1990). Immortalization of murine microglial cells by a v-raf/v-myc carrying retrovirus. J Neuroimmunol.

[22] Li Y, Liu L, Barger S, Griffin W (2003). Interleukin-1 mediates pathological effects of microglia on tau phosphorylation and on synaptophysin synthesis in cortical neurons through a p38-MAPK pathway. J Neurosci.

[23] Yu W, Go L, Guinn B, Fraser P, Westaway D, McLaurin J (2002). Phenotypic and functional changes in glial cells as a function of age. Neurobiol Aging.

[24] Vorhees C, Williams M (2006). Morris water maze: procedures for assessing spatial and related forms of learning and memory. Nat Protoc.

[25] ONeill L, Kaltschmidt C (1997). NF-kappa B: a crucial transcription factor for glial and neuronal cell function. Trends Neurosci.

[26] Hwang J, Hwang H, Lee H, Suk K (2010). Microglia signaling as a target of donepezil. Neuropharmacology.

[27] Huang D, Wujek J, Kidd G, He T, Cardona A, Sasse M, Stein E, Kish J, Tani M, Charo I, Proudfoot A, Rollins B, Handel T, Ransohoff R (2005). Chronic expression of monocyte chemoattractant protein-1 in the central nervous system causes delayed encephalopathy and impaired microglial function in mice. FASEB J.

[28] Klegeris A, Maguire J, McGeer P (2004). S- but not R-enantiomers of flurbiprofen and ibuprofen reduce human microglial and THP-1 cell neurotoxicity. J Neuroimmunol.

[29] Weggen S, Eriksen J, Das P, Sagi S, Wang R, Pietrzik C, Findlay K, Smith T, Murphy M, Butler T, Kang D, Marquez-Sterling N, Golde T, Koo E (2001). A subset of NSAIDs lower amyloidogenic A beta 42 independently of cyclooxygenase activity. Nature.

[30] Heneka M, Sastre M, Dumitrescu-Ozimek L, Hanke A, Dewachter I, Kuiperi C, O’Banion K, Klockgether T, Van Leuven F, Landreth G (2005). Acute treatment with the PPAR gamma agonist pioglitazone and ibuprofen reduces glial inflammation and A beta 1–42 levels in APPV717I transgenic mice. Brain.

[31] Van Dam D, Coen K, De Deyn P (2010). Ibuprofen modifies cognitive disease progression in an Alzheimer’s mouse model. J Psychopharmacol.

[32] Pan X, Chen X, Zhu Y, Chen L, Zhang J, Huang T, Ye Q, Huang H (2009). Tripchlorolide protects neuronal cells from microglia-mediated beta-amyloid neurotoxicity through inhibiting NF-kappa B and JNK signaling. Glia.

[33] Guha M, Mackman N (2001). LPS induction of gene expression in human monocytes. Cell Signal.

[34] Liu X, Pan L, Wang X, Gong Q, Zhu Y (2012). Leonurine protects against tumor necrosis factor-alpha-mediated inflammation in human umbilical vein endothelial cells. Atherosclerosis.

[35] Kondo S, Sauder D (1997). Tumor necrosis factor (TNF) receptor type 1 (p55) is a main mediator for TNF-alpha-induced skin inflammation. Eur J Immunol.

[36] Takeuchi O, Hoshino K, Kawai T, Sanjo H, Takada H, Ogawa T, Takeda K, Akira S (1999). Differential roles of TLR2 and TLR4 in recognition of gram-negative and gram-positive bacterial cell wall components. Immunity.

[37] McLarnon J, Ryu J (2008). Relevance of A beta(1–42) intrahippocampal injection as an animal model of inflamed Alzheimer’s disease brain. Curr Alzheimer Res.

[38] Fakhfouri G, Ahmadiani A, Rahimian R, Grolla A, Moradi F, Haeri A (2012). WIN55212-2 attenuates amyloid-beta-induced neuroinflammation in rats through activation of cannabinoid receptors and PPAR-gamma pathway. Neuropharmacology.

[39] Yamada K, Nabeshima T (2000). Animal models of Alzheimer’s disease and evaluation of anti-dementia drugs. PharmacolTher.

[40] Walker D, Lue L, Beach T (2001). Gene expression profiling of amyloid beta peptide-stimulated human post-mortem brain microglia. Neurobiol Aging.

[41] Choi M, Kim E, Hahn H, Dal Nam K, Yang S, Choi S, Kim T, Cho S, Huh J (2007). Protective effect of benzothiazole derivative KHG21834 on amyloid beta-induced neurotoxicity in PC12 cells and cortical and mesencephalic neurons. Toxicology.

[42] Gong Q, Pan L, Liu X, Wang Q, Huang H, Zhu Y (2011). S-propargyl-cysteine (ZYZ-802), a sulphur-containing amino acid, attenuates beta-amyloid-induced cognitive deficits and pro-inflammatory response: involvement of ERK1/2 and NF-kappa B pathway in rats. Amino Acids.

